# Fourier Transform Infrared Spectroscopy (FT-IR) and Simple Algorithm Analysis for Rapid and Non-Destructive Assessment of Developmental Cotton Fibers

**DOI:** 10.3390/s17071469

**Published:** 2017-06-22

**Authors:** Yongliang Liu, Hee-Jin Kim

**Affiliations:** 1 Cotton Structure & Quality Research Unit, United States Department of Agriculture (USDA), Agricultural Research Service (ARS), New Orleans, LA 70124, USA; 2 Cotton Fiber Bioscience Research Unit, United States Department of Agriculture (USDA), Agricultural Research Service (ARS), New Orleans, LA 70124, USA; HeeJin.kim@ars.usda.gov

**Keywords:** Fourier transform infrared spectroscopy, attenuated total reflection, cellulose, fiber secondary cell wall biosynthesis, principal component analysis, algorithm analysis

## Abstract

With cotton fiber growth or maturation, cellulose content in cotton fibers markedly increases. Traditional chemical methods have been developed to determine cellulose content, but it is time-consuming and labor-intensive, mostly owing to the slow hydrolysis process of fiber cellulose components. As one approach, the attenuated total reflection Fourier transform infrared (ATR FT-IR) spectroscopy technique has also been utilized to monitor cotton cellulose formation, by implementing various spectral interpretation strategies of both multivariate principal component analysis (PCA) and 1-, 2- or 3-band/-variable intensity or intensity ratios. The main objective of this study was to compare the correlations between cellulose content determined by chemical analysis and ATR FT-IR spectral indices acquired by the reported procedures, among developmental Texas Marker-1 (TM-1) and immature fiber (*im*) mutant cotton fibers. It was observed that the *R* value, *CI*_IR_, and the integrated intensity of the 895 cm^−1^ band exhibited strong and linear relationships with cellulose content. The results have demonstrated the suitability and utility of ATR FT-IR spectroscopy, combined with a simple algorithm analysis, in assessing cotton fiber cellulose content, maturity, and crystallinity in a manner which is rapid, routine, and non-destructive.

## 1. Introduction

**Figure 1:**
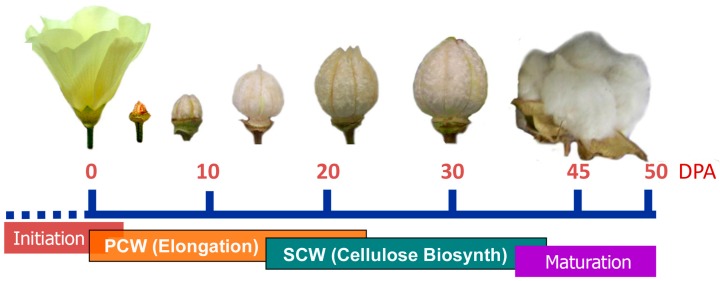
Schematic of general cotton fiber growth at various days post anthesis (DPA).

As one of the most important and widely grown crops in the world, cotton is a well-traded agricultural commodity primarily for its naturally produced textile fiber [1]. Commercial cotton fibers are harvested from cotton plants. In biological terms, cotton fibers are the dried cell walls of formerly living cells. They initiate from an ovary of the flower and grow into a mature seed-containing cotton boll within approximately 1.5~2 months. Fiber growth consists of four overlapping but distinctive phases: initiation, primary cell wall (PCW) formation for fiber elongation, secondary cell wall (SCW) biosynthesis for cellulose deposition and cell wall thickening, and maturation [2,3]. The day of flowering is referred to as anthesis, and the term “days post anthesis” (DPA) is commonly used to describe the cotton fiber growth (Figure 1). The fiber cells initiate at 0 DPA and then elongate to reach a fiber length of 22~35 mm within 20 to 25 DPA. The secondary cell wall synthesis starts around 15 to 22 DPA and continues for an additional 30 to 40 days until the maturation phase, when the fibers dehydrate and collapse into flattened and twisted ribbons. The apparent cellulose amount in cotton fibers increases with cotton fiber growth, leading to significant differences in chemical, physical, and compositional attributes during cotton fiber development. Over the years, diversified and well-defined fiber testing methods, such as high volume instrument (HVI) and advanced fiber information system (AFIS), have been developed to reflect these changes routinely in the cotton industry [4].

Mature fibers are composed mostly of cellulose (88.0–96.5%), followed by such non-cellulosic constituents as proteins (1.0–1.9%), waxes (0.4–1.2%), pectins (0.4–1.2%), inorganics (0.7–1.6%), and other substances (0.5–8.0%) [5]. Analysis of cell wall compositions of fibers from the early stages of elongation through the period of the SCW formation was achieved by a set of extraction, separation, and isolation steps, prior to chemical and instrumental determination of targeted components [6,7,8,9].

Cotton fiber cellulose is not easily dissolved in most solvent, and the extraction and separation process in conventional methods experience significant drawbacks that include the tedious procedures of optimal solvent and temperature selection as well as extracted specimen identification. As the need for more rapid approaches inceases, a number of available analytical techniques, including Fourier transform infrared (FT-IR) spectroscopy, differential scanning calorimeter (DSC), thermogravimetric analysis (TGA), and pyrolysis-gas chromatography/mass spectroscopy (GC/MS) methods [10,11,12,13,14,15], have been applied to identify cellulose and non-cellulose components in cottons. These measurements provide clear evidence of various components in developing cotton fibers through the onset of SCW synthesis. For example, the appearance of FT-IR bands at 1733 cm^−1^ (C=O stretching originating from esters or amides) and 1534 cm^−1^ (NH_2_ deformation corresponding to proteins or amino acids) reflects the presence of such non-cellulosic components as esters and proteins in cotton fibers [11,13]. Among these techniques, the ATR FT-IR spectroscopy method has evolved as an important alternative to examine cotton fiber development, because it requires minimal sample preparation by ATR sampling on a small bundle of cotton fibers as little as 0.5 mg, permits routine analysis rapidly and non-destructively, and is easy to operate [10,11,13,14,15,16,17,18,19,20].

Different spectral interpretation approaches have been applied to acquire useful information from FT-IR measurement. It includes the direct use of 1-band intensity, the estimation of 2- or 3-band intensity ratios, and the implementation of a chemometrical or multivariate tool known as principal component analysis (PCA) [10,11,13,14,15,16,17,18,19,20,21]. The results have indicated that both simple algorithms and PCA patterns can be used to monitor the transition from PCW to SCW biosyntheses in cotton fibers. Interestingly, simple algorithms were observed to have the ability to detect the subtle discrepancies in fibers older than 25 DPA among respective fibers grown in planta or in culture [17]. In the current work, we performed the PCA and simple algorithm examination of ATR FT-IR spectra representing the developmental immature fiber (*im*) mutant fibers and its near-isogenic wild type Texas Marker-1 (TM-1) fibers, and compared the correlations between cellulose content determined by conventional chemical analysis and respective ATR FT-IR spectral responses acquired by various spectral interpretation strategies. Notably, simple algorithm analysis of ATR FT-IR spectra has been developed to estimate fiber cellulose maturity and crystallinity simultaneously in developing cottons [14,15,16,17].

## 2. Materials and Methods

### 2.1. Texas Marker-1 (TM-1) and Immature Fiber (im) Mutant Fibers

Two cotton near-isogenic lines (NILs), TM-1 and *im*, were grown side by side in a field of USDA-ARS (New Orleans, LA, USA) in 2011. At day of post anthesis (DPA), cotton flowers were tagged. Two biological replicates of cotton bolls were taken from different cotton plants at 10, 17, 24, 28, 33, 37 and 44 DPA. Fibers at each DPA were collected from 10 to 30 bolls in fifty plants for each biological replication by manually removing the seeds, prior to drying in 40 °C incubator. The soil type was Aquent dredged over alluvium in an elevated location to provide adequate drainage. Harvested fibers were kept in a dark storage room with a constant temperature (23 ± 1 °C) and relative humidity (50 ± 10%), and their ATR FT-IR spectra were collected in March 2014. The ~2.5 years gap between the harvest of fibers and the collection of FT-IR spectra was due to the beginning of collaboration between two authors in March 2014.

### 2.2. Cellulose Content

Fiber cellulose content at each developmental stage was measured by the modified Updegraff method [22]. Briefly, 10 mg of cut fibers were placed into 5 mL of reacti-vials. Non-cellulosic materials in fibers were hydrolyzed with acetic-nitric reagent (a mixture of 73% acetic acid, 9% nitric acid and 18% water). The remaining cellulose was hydrolyzed with 67% sulfuric acid (*v*/*v*) and measured by a colorimetric assay with anthrone and by the use of Avicel PH-101 (FMC, Rockland, ME, USA) as a cellulose standard. The average cellulose content for each fiber was obtained from three replications. It took at least 2 days to measure cellulose content for each sample mostly due to slow hydrolysis process of fiber cellulose component.

### 2.3. ATR FT-IR Spectral Collection and Data Analysis

All fibers were scanned by an FTS 3000MX FT-IR spectrometer (Varian Instruments, Randolph, MA, USA) equipped with a ceramic source, KBr beam splitter, and deuterated triglycine sulfate (DTGS) detector and attenuated total reflection (ATR) attachment. The ATR sampling device utilized a DuraSamplIR single-pass diamond-coated internal reflection accessory (Smiths Detection, Danbury, CT, USA), and a consistent contact pressure was applied by way of a stainless steel rod and an electronic load display. During the data collection, cautions were taken to make sure that the window (2 mm in diameter) of the ATR sampling devise was covered completely by fiber samples. At least five measurements for individual fiber samples, by re-sampling at different locations across entire sample, were collected over the range of 4000–600 cm^−1^ at 4 cm^−1^ and 16 co-added scans. All spectra were given in absorbance units and no ATR baseline correction was applied.

Importing the spectra to the GRAMS IQ application in Grams/AI (Version 9.1, Thermo Fisher Scientific, Waltham, MA, USA), the mean spectrum was taken for each sample and then was smoothed with a Savitzky–Golay function (polynomial = 2 and points = 11). The spectra were normalized by dividing the intensity of the individual band in the 1800–600 cm^−1^ region with the average intensity in this 1800–600 cm^−1^ region, and subsequent PCA characterization was performed in the 1800–600 cm^−1^ IR region, with mean centering (MC) and Savitzky–Golay first-derivative (2 degrees and 13 points) spectral pretreatment, as well as with the leave-one-out cross-validation method. With the use of the Grams/AI program, integrated intensities of 4 bands at 2900, 1372, 895, and 664 cm^−1^ were estimated in the respective ranges of 3000 to 2800, 1410 to 1290, 910 to 875, and 684 to 650 cm^−1^ from normalized spectra. Separately, the spectral set was loaded into Microsoft Excel 2007 to execute simple algorithm analysis.

## 3. Results and Discussion

### 3.1. DPA-Dependent Cellulose Content of Developmental Fibers

**Figure 2:**
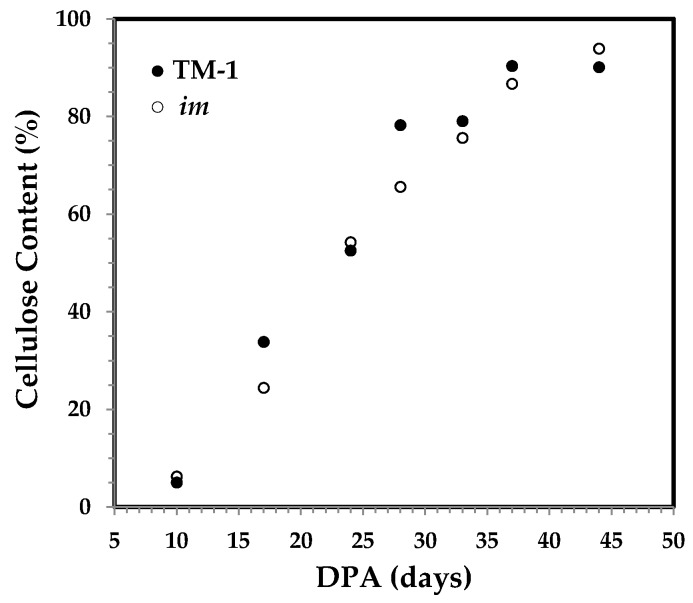
Cellulose content against DPA for developmental Texas Marker-1 (TM-1) (●) and immature fiber (*im*) (○) fibers by the modified Updegraff method [22].

The DPA-dependent cellulose content between the two sets of developmental fibers in Figure 2 shows a similar increasing pattern between the two types of fibers at various developmental stages. For either TM-1 or *im* fiber, cellulose content increases rapidly and linearly from 10 to 37 DPA, as expected.

### 3.2. ATR FT-IR Spectral Characteristics of Developmental TM-1 Cotton Fibers

**Figure 3:**
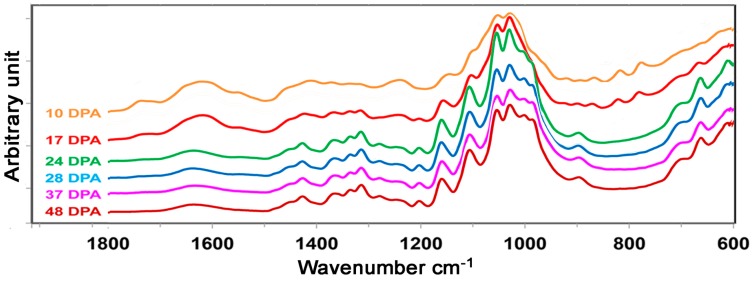
Attenuated total reflection Fourier transform infrared (ATR FT-IR) spectral response of TM-1 fibers to various DPA.

With fiber DPA progressing (Figure 1), apparent spectral intensity increases or decreases in the mid infrared (mid-IR) region of 1800–600 cm^−1^ are anticipated for these developmental TM-1 cotton fibers (Figure 3). A thorough examination of ATR FT-IR spectral feature reveals that TM-1 fiber exhibits a nearly identical spectral pattern to *im* fiber at same developmental point. TM-1 fibers and *im* fibers, alike, demonstrate growth results in the dominant production of major common chemical component in cotton fibers, cellulose. Spectral intensity changes in Figure 3 are in good agreement with those reported earlier [11,13,17], and characteristic band assignments have been summarized in Table 1. Briefly, the vibration at 1740 cm^−1^ is assigned to the C=O stretching mode of carbonyl groups due to lipids, and a broad band centered at 1620 cm^−1^ is mainly attributed to the OH bending mode of adsorbed water. Bands in the region of 1500–1200 cm^−1^ represent the contributions of both CH_2_ deformations and C–O–H bending vibrations, and those bands in the 1200–900 cm^−1^ region originate from the coupling modes of C–O and C–C vibrations. The bands between 800 and 700 cm^−1^ are likely due to two crystal forms (*I*_α_ and *I*_β_) of cotton cellulose. In addition, there are intense absorptions between the 3600 and 2750 cm^−1^ regions that are assignable to the O–H and C–H stretching vibrations (not shown).

A comparison of the increasing or decreasing intensity of these bands between shorter DPA (for example, 10 DPA) and longer DPA (for example, 37 DPA) fibers is tabulated in Table 1. In general, intensities of the bands at 1740, 1620, 1545, 1405, and 1236 cm^−1^ as well as those in the 850–750 cm^−1^ region decrease, while those at 1425, 1365, 1335, 1315, 1200, 1158, 1104, 1055, 1028, 985, 895 and 662 cm^−1^ increase. Subjective interpretation of these spectra in Figure 3 cannot be applied to compare or assess the degree of fiber secondary wall biosynthesis in a semi-quantitative way.

### 3.3. Correlation between Cellulose Content and ATR FT-IR Spectral Response of Developmental Fibers

#### 3.3.1. PC1 Score and the Correlation with Cellulose Content 

**Figure 4:**
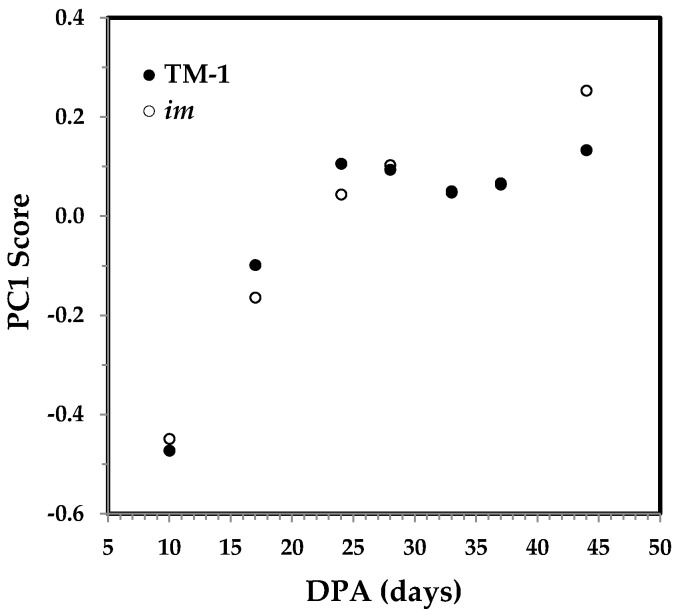
First principal component (PC1) score against DPA from ATR FT-IR spectra of developmental TM-1 (●) and *im* (○) fibers.

As usual, PCA were performed to understand the similarity or dissimilarity of ATR FT-IR spectra that are indicative of fiber growth for TM-1 and *im* fibers. The plot of the first principal component (PC1) score vs. DPA in Figure 4 provides a good visualization of the sample distribution among two sets of fibers. The first two PCs accounted for 89.5% of the total variation, with the PC1 explaining 80.4% of the variation. For both TM-1 and *im* fibers, PC1 scores increase rapidly between 10 and 24 DPA before reaching the relatively constant PC1 score. It implies that the 10 DPA fibers are composed of PCW constituents, whereas the 24 DPA fibers consist of SCW components. The observation is consistent with accumulated knowledge suggesting that elongating fibers at 10 DPA contain no SCW cellulose whereas thickening fibers at 24 DPA are composed of more SCW cellulose. Notably, the PC1 scores are nearly independent of fiber DPA when DPA is more than 25 days, suggesting that the overall contributions from spectral intensity variations in the 1800–600 cm^−1^ region during this period of fiber maturation are probably insignificant.

**Figure 5:**
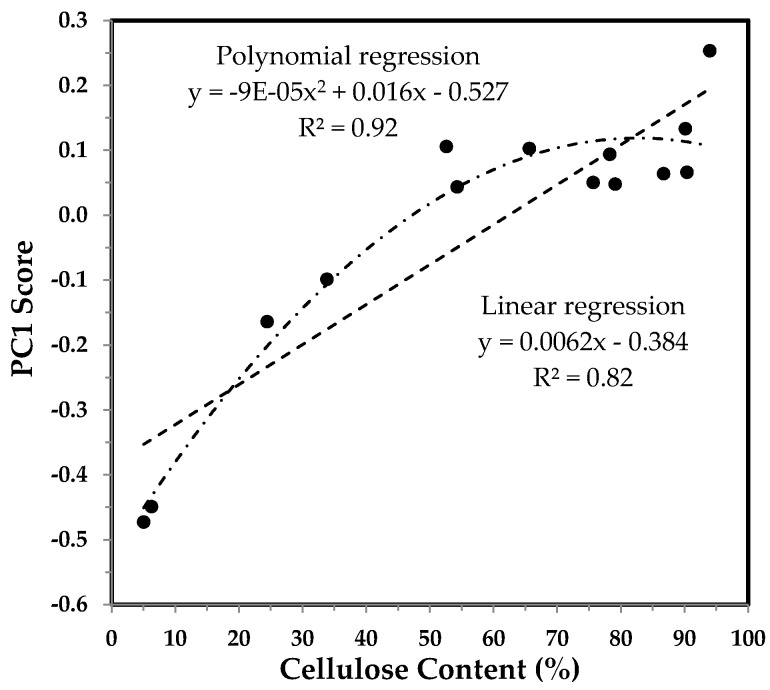
PC1 score vs. cellulose content of developmental TM-1 and *im* fibers. Linear and polynomial (with the order of 2) regressions were applied to all fiber samples.

The plot of relating cellulose content to PC1 score for developmental TM-1 and *im* fibers in Figure 5 offers a better visualization of relationship between two parameters. In general, cellulose content increases with PC1 score up to 24 DPA by referring to the Figure 4. Remarkably, the correlation between the two parameters could be described more accurately by a polynomial (with the order of 2) regression than by a linear regression, as shown in Figure 5. It implies that cellulose content has a weak linear relationship with PC1 score.

#### 3.3.2. IR maturity and Crystallinity and the Correlation with Cellulose Content 

**Figure 6:**
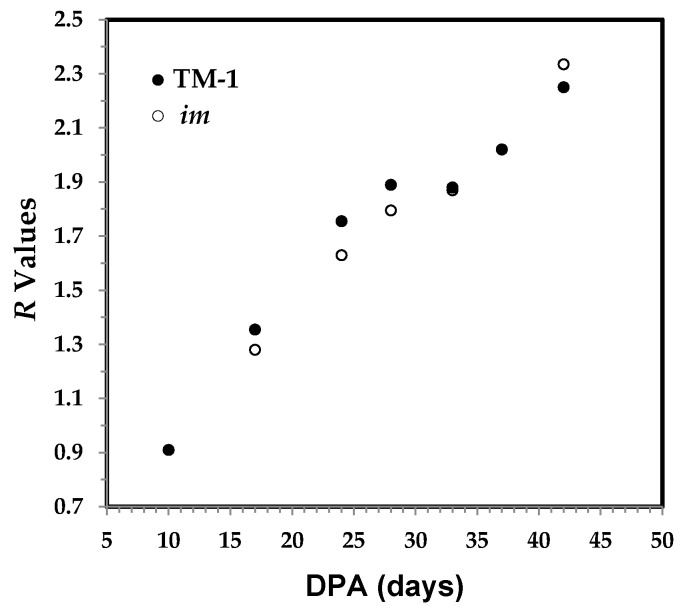
*R* value against DPA from ATR FT-IR spectra of developmental TM-1 (●) and *im* (○) fibers.

Compared to the PCA approach that utilized a number of 624 datapoints or variables (from the 1800 to 600 cm^−1^ IR region with 1.949 cm^−1^ interval in this study), previously proposed 3-band based ratios or algorithms were assessed on the same spectral dataset. The three bands at 1800, 1315, and 1236 cm^−1^ were used to assess the ratio *R* values and these values were found to be related with fiber growth [17]. The ratio *R* values were assessed by the algorithm of *R* = (A_1315_ − A_1800_)/(A_1236_ − A_1800_), where A_1800_, A_1315_, and A_1236_ represent the respective band intensities centered at 1800, 1315, and 1236 cm^−1^ [17]. The 1800 cm^−1^ band was offset to be zero in intensity subjectively. The 1315 cm^−1^ band arises from the CH_2_ wagging mode that is increasing in intensity with DPA, while the 1236 cm^−1^ band assigned to O–H/N–H deformation decreases in intensity. As shown in Figure 6, the *R* value increases along with the DPA, both directly and obviously. The pattern suggests that this algorithm is capable of reflecting the continuous cellulose production in fibers older than 24 DPA, which was not apparent from the PC1 score of the PCA result in Figure 4.

**Figure 7:**
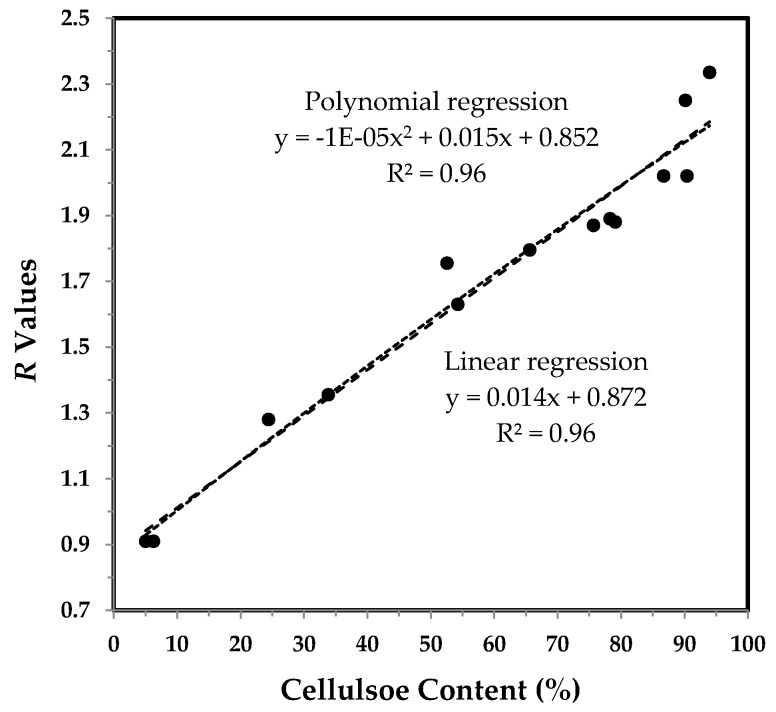
*R* value vs. cellulose content of developmental TM-1 and *im* fibers. Linear and polynomial (with the order of 2) regressions were applied to all fiber samples.

Unlike the trend in Figure 5, there is no visual disparity in Figure 7 between a polynomial regression and a linear regression. A strong linear correlation (*R*^2^ = 0.96) is within the expectation since two independent methods should be consistent in representing cotton fiber growth. This result demonstrates that the ATR FT-IR technique, along with this simple algorithm, can be used for monitoring fiber cellulose content and fiber maturity, rapidly and non-destructively.

**Figure 8:**
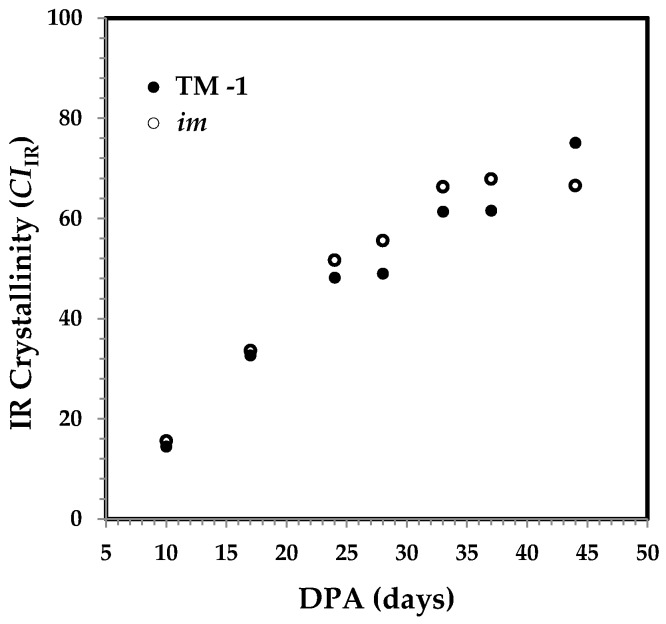
IR crystallinity (*CI*_IR_) against DPA from ATR FT-IR spectra of developmental TM-1 (●) and *im* (○) fibers.

A further interest was to estimate IR crystallinity (*CI*_IR_) by using the respective IR bands at 708, 730, and 800 cm^−1^ [15,16,17]. Two bands at 708 and 730 cm^−1^ are assignable to *I*_β_ cellulose in crystalline part and to *I*_α_ cellulose in amorphous part, respectively, whereas the 800 cm^−1^ band was used to offset in intensity subjectively. Plot of *CI*_IR_ against DPA in Figure 8 reveals a steady *CI*_IR_ increase for each fiber set, as anticipated. The general trend in Figure 8 is similar to that in Figure 6 more so than that which is in Figure 4, when examining the *CI*_IR_ values for the fibers older than 24 DPA.

**Figure 9:**
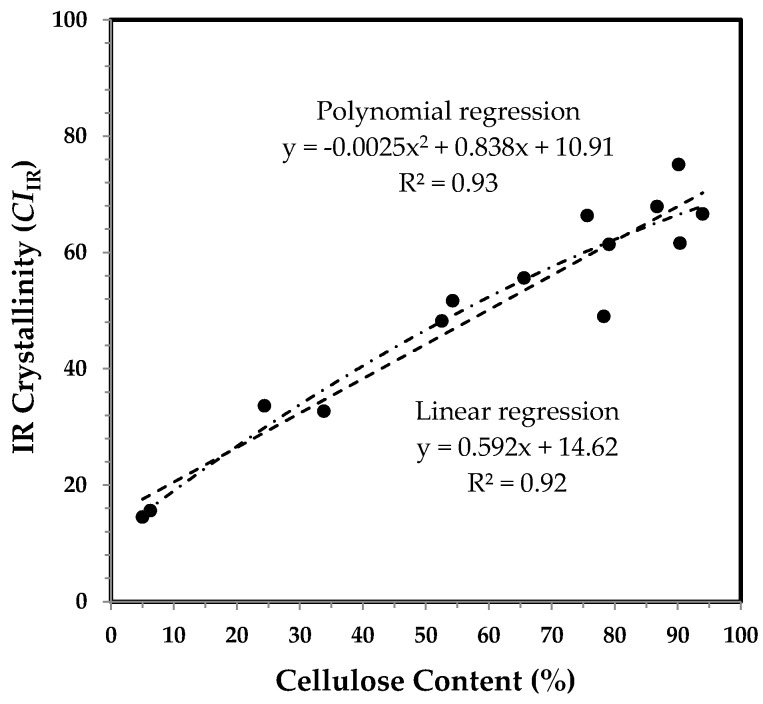
**Figure 9**. IR crystallinity (*CI*_IR_) vs. cellulose content of developmental TM-1 and *im* fibers. Linear and polynomial (with the order of 2) regressions were applied to all fiber samples.

Figure 9 shows a high linear relationship (*R*^2^ = 0.92) between the degree of *CI*_IR_ and cellulose content. There is an insignificant difference in Figure 9 between a polynomial regression and a linear regression, and the pattern in Figure 9 is much closer to that in Figure 7, than that which is in Figure 5. A strong linear correlation (*R*^2^ = 0.92) is much expected, because cotton fiber crystallinity increases with cotton fiber growth [14,16,17,18]. This result verifies that the ATR FT-IR technique, along with this simple algorithm, can also be used for assessing fiber crystallinity, rapidly and non-destructively.

#### 3.3.3. The 1372 and 2900 cm^−1^ Band Based IR Crystallinity and the Correlation with Cellulose Content 

**Figure 10:**
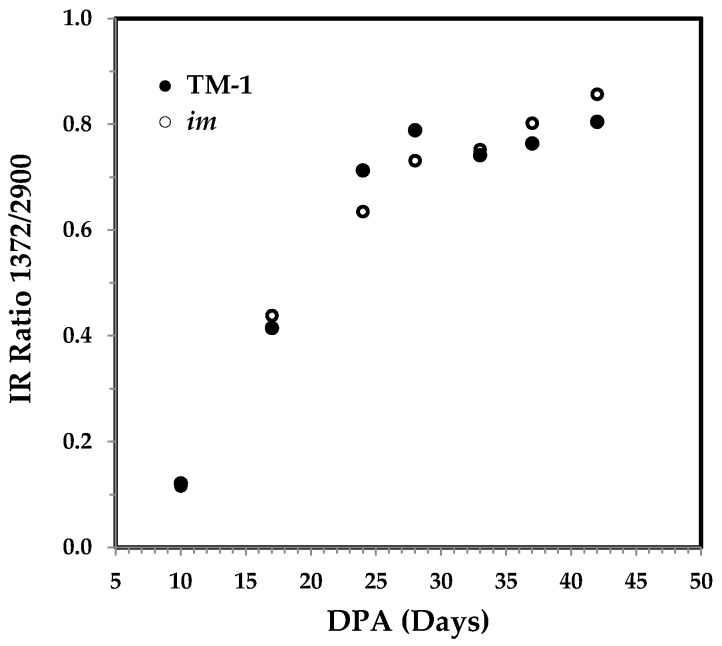
IR Ratio 1372/2900 against DPA from ATR FT-IR spectra of developmental TM-1 (●) and *im* (○) fibers. The ratios were calculated from integrated intensities of two bands at 1372 and 2900 cm^−1^.

Two bands at 1372 and 2900 cm^−1^ based IR Ratio 1372/2900 have been reported to indirectly estimate the percentage crystallinity in cellulose materials [14,21]. The 1372 cm^−1^ band is attributed to the C–H bending vibration, while the 2900 cm^−1^ band is assigned to the C–H stretching mode. As shown in Figure 10, the IR ratio 1372/2900 elevates rapidly from 10 to 28 DPA, and is consistent with different cotton varieties reported by other researchers [14]. The pattern in Figure 10 resembles the PC1 score of PCA representation in Figure 4, other than those in Figure 6 and Figure 8.

**Figure 11:**
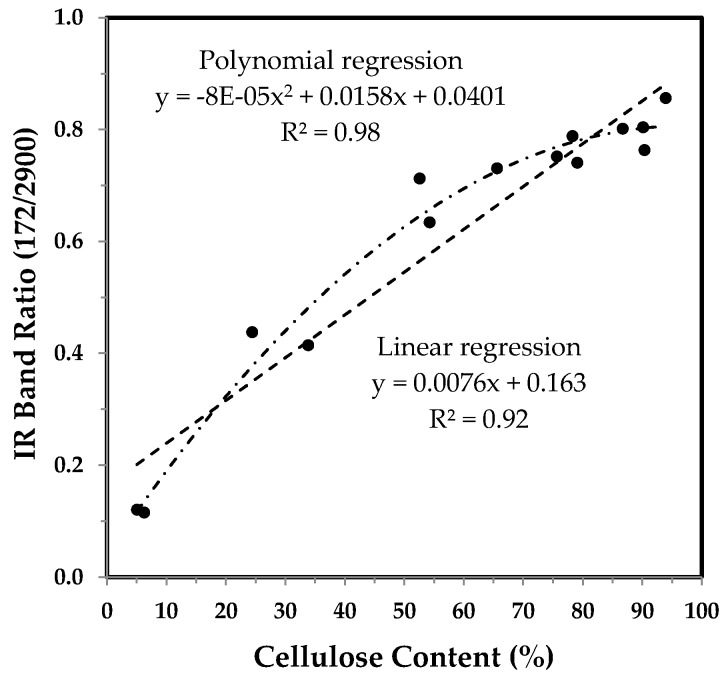
IR Ratio 1372/2900 vs. cellulose content of developmental TM-1 and *im* fibers. Linear and polynomial (with the order of 2) regressions were applied to all fiber samples.

Much like the tendency in Figure 5, cellulose content increases with the IR Ratio 1372/2900 in the manner of a polynomial curve over a linear curve (Figure 11).

#### 3.3.4. Integrated Intensity of the 895 cm^−1^ Band and the Correlation with Cellulose Content 

**Figure 12:**
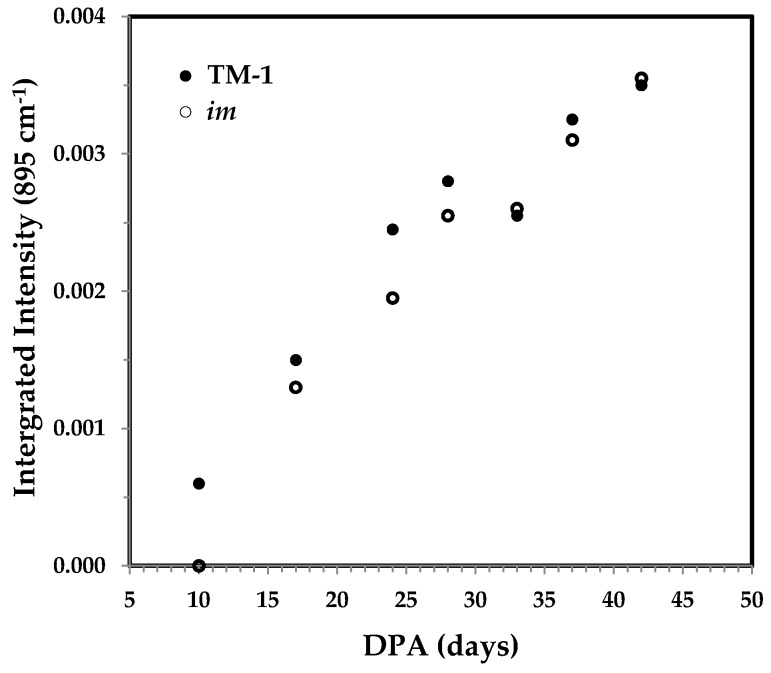
Integrated intensity of the 895 cm^−1^ band against DPA from normalized ATR FT-IR spectra of developmental TM-1 (●) and *im* (○) fibers.

The band at 895 cm^−1^ has been assigned to the β-glycosidic linkage in cellulose, and its integrated intensity was found to be linearly correlated with the percentage of cellulose [13] and of crystallinity [14]. Slightly differing from the relationship in a previous report [13], Figure 12 indicates an obvious linear response of integrated intensity of the 895 cm^−1^ band to fiber growth.

**Figure 13:**
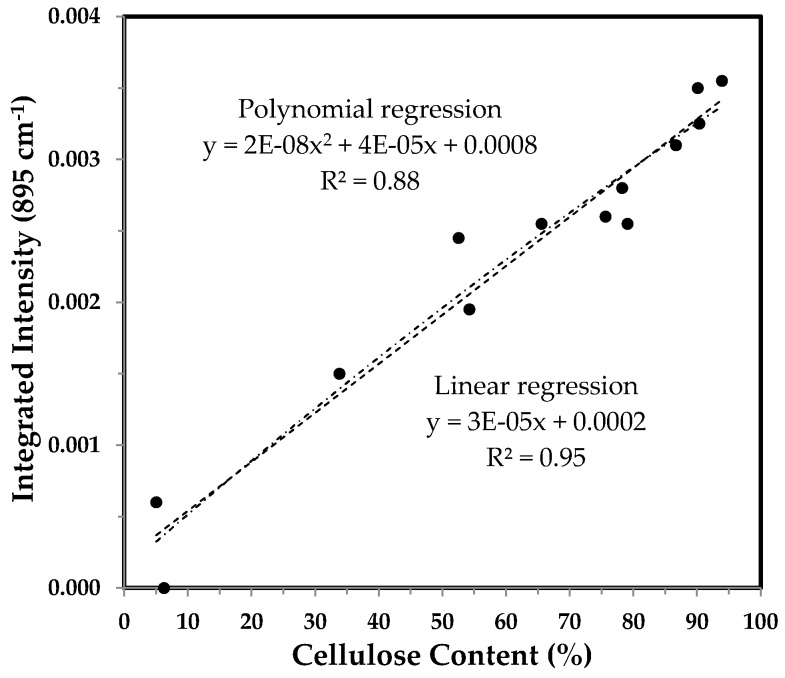
Integrated intensity of the 895 cm^−1^ band vs. cellulose content of developmental TM-1 and *im* fibers. Linear and polynomial (with the order of 2) regressions were applied to all fiber samples.

A great linear relationship (*R*^2^ = 0.95) is observed between the integrated intensity of the 895 cm^−1^ band and cellulose content (Figure 13). The pattern in Figure 13 is identical to those in Figure 7 and Figure 9.

#### 3.3.5. Integrated Intensity of the 664 cm^−1^ Band and the Correlation with Cellulose Content 

**Figure 14:**
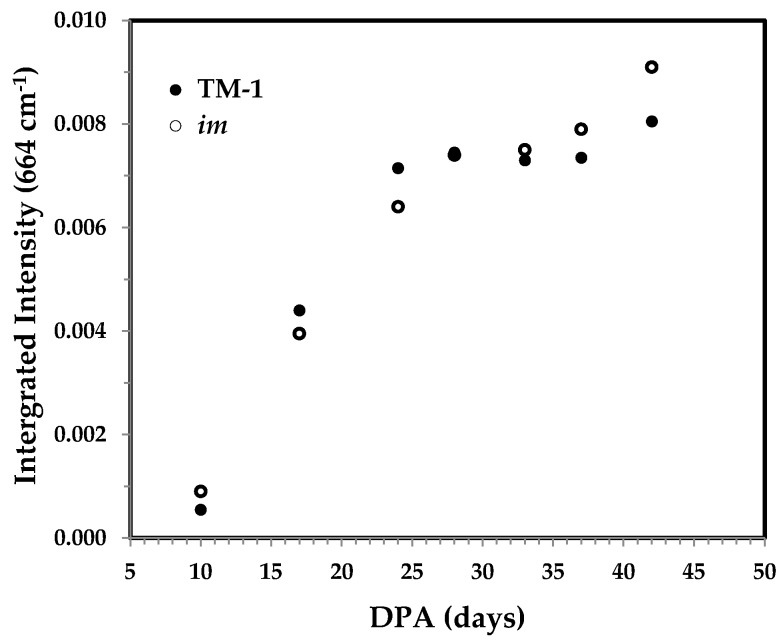
Integrated intensity of the 664 cm^−1^ band against DPA from normalized ATR FT-IR spectra of developmental TM-1 (●) and *im* (○) fibers.

The band at 664 cm^−1^ has been ascribed to OH out-of-plane bending mode [13,14]. Its integrated intensity had a good linear relationship with cellulose content [13] and the percentage of crystallinity in cotton fibers [14]. The trend in Figure 14 is much similar to the PC1 score of PCA result in Figure 4 and the IR Ratio 1372/2900 in Figure 10, and also agrees with that for other cotton varieties [13].

**Figure 15:**
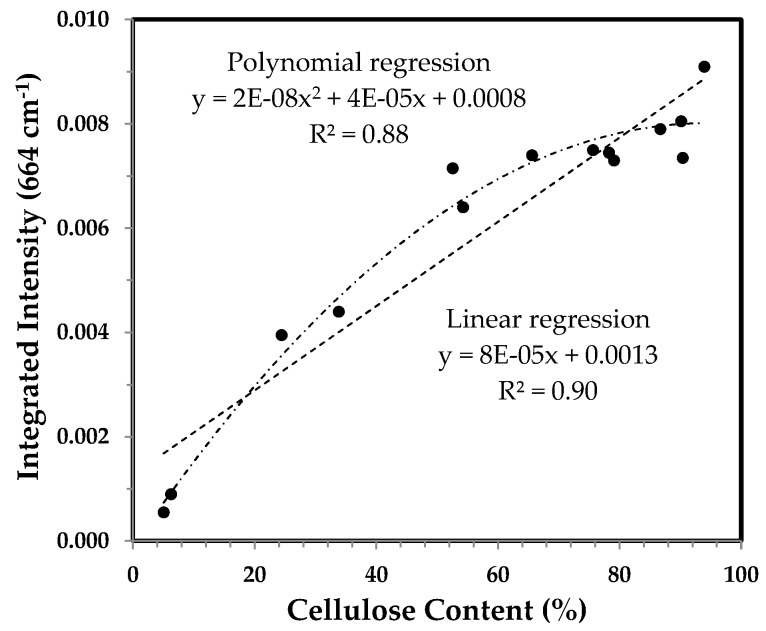
Integrated intensity of the 664 cm^−1^ band vs. cellulose content of developmental TM-1 and *im* fibers. Linear and polynomial (with the order of 2) regressions were applied to all fiber samples.

Figure 15, being dissimilar from the patterns in Figure 7, Figure 9 and Figure 13, reveals a similar tendency to that which is in Figure 5 and Figure 11, when relating the integrated intensity of the 664 cm^−1^ band to cellulose content.

## 4. Conclusions

The present study demonstrates the great potential of ATR FT-IR spectroscopy, in conjunction with simple algorithm analysis, in sensing the cellulose deposition during cotton fiber development. Upon comparing the correlations between cellulose content determined by assay analysis and the ATR FT-IR spectral response acquired by multivariate PCA and the proposed simple algorithms, it has become evident that the *R* value, *CI*_IR_, and the integrated intensity of the 895 cm^−1^ band can be utilized to estimate cotton fiber cellulose content, maturity and crystallinity, in a rapid, routine, and non-destructive manner.

## Figures and Tables

**Table 1:** Characteristic ATR FT-IR spectral bands of shorter and longer DPA fibers [11,13,17] *.

10 DPA Fiber	37 DPA Fiber	Band Assignment
1740 (s)	1740 (w)	C=O stretching
1620 (s)		HOH bending of adsorbed water + amide I
	1620 (w)	HOH bending of adsorbed water
1545 (s)	1545 (w)	Amide II
1425 (w)	1425 (s)	CH_2_ scissoring
1405 (s)	1405 (w)	O–H deformation
1365 (w)	1365 (s)	C–H bending
1335 (w)	1335 (s)	CH_2_ wagging
1315 (w)	1315 (s)	CH_2_ wagging
1236 (s)	1236 (w)	O–H deformation or N–H deformation
1200 (w)	1200 (s)	C–O stretching
1158 (w)	1158 (s)	C–O–C stretching
1104 (w)	1104 (s)	C–O stretching
1055 (w)	1055 (s)	C–O stretching
1028 (w)	1028 (s)	C–O stretching
985 (w)	985 (s)	C–O stretching
895 (w)	895 (s)	β-glycosidic linkage
662 (w)	662 (s)	O–H out-of-plane bending

* Parentheses indicate the relative ATR FT-IR spectral intensities between 10 and 37 DPA fibers: s = strong, w = weak.
